# Mechanical Properties of High- and Low-Fusing Zirconia Veneering Ceramics Fired on Different Trays and Substrates

**DOI:** 10.3390/ma17102261

**Published:** 2024-05-10

**Authors:** Moritz Hoffmann, Andrea Coldea, Mustafa Borga Dönmez, John Meinen, Bogna Stawarczyk

**Affiliations:** 1 Department of Prosthetic Dentistry, Dental School, Ludwig-Maximilians-Universität München, 80336 Munich, Germany; moritz.hoffmann@med.uni-muenchen.de (M.H.); andrea.coldea@med.uni-muenchen.de (A.C.); john.meinen@med.uni-muenchen.de (J.M.); bogna.stawarczyk@med.uni-muenchen.de (B.S.); 2 Department of Reconstructive Dentistry and Gerodontology, School of Dental Medicine, University of Bern, 3010 Bern, Switzerland; 3 Department of Prosthodontics, Faculty of Dentistry, Istinye University, Istanbul 34010, Turkey

**Keywords:** firing tray, flexural strength, Martens’ parameters, shrinkage, veneering ceramic

## Abstract

This study aimed to evaluate the effect of ceramic type, firing tray, and firing substrate on the density, shrinkage, biaxial flexural strength, Martens’ hardness, and elastic indentation modulus of zirconia veneering ceramics. Disk-shaped specimens were fabricated from a high-fusing (HFZ) and a low-fusing (STR) zirconia veneering ceramic. These specimens were then divided into 10 groups according to firing trays (round, small honeycomb-shaped, cordierite [RSC]; round, large honeycomb-shaped, aluminum oxide [RLA]; rectangular, plane, silicon nitride [RCPS]; round, plane, silicon nitride [RPS]; and rectangular, plane, calcium silicate [RCPC]) and firing substrates (firing cotton and platinum foil) used (n = 12). The density, shrinkage, biaxial flexural strength, Martens’ hardness, and indentation modulus were measured, and analyzed with generalized linear model analysis (α = 0.05). The interaction between the ceramic type and firing substrate affected density (*p* < 0.001), and the other outcomes were affected by the interaction among all main factors (*p* ≤ 0.045). Higher density was observed with HFZ or platinum foil (*p* ≤ 0.007). RSC and RLA led to a higher density than RCPS within HFZ and led to the lowest density within STR (*p* ≤ 0.046). STR had a higher shrinkage (*p* < 0.001). RSC mostly led to a lower shrinkage of HFZ (*p* ≤ 0.045). The effect of ceramic type and firing substrates on the biaxial flexural strength, Martens’ hardness, and indentation modulus was minimal while there was no clear trend on the effect of firing tray on these properties. Ceramic type, firing tray, and firing substrate affected the mechanical properties of the tested zirconia veneering ceramics. Firing the tested zirconia veneering ceramics over a round and small honeycomb-shaped cordierite firing tray with firing cotton mostly led to improved mechanical properties.

## 1. Introduction

Zirconia, which has three crystallographic structures, monoclinic, tetragonal, and cubic [1], has become an essential part of dentistry and started to replace metal-fused porcelain prostheses, given the esthetic demands of patients, which have significantly increased in recent years [2,3]. Even though zirconia’s superior mechanical properties and biocompatibility have broadened its range of applications [4], its monolithic use is limited by its inherent opaqueness [5]. New-generation zirconia offers improved translucency with increased cubic crystalline content [1]; however, veneering ceramic is still needed for more esthetic prostheses [6]. A zirconia prosthesis can be veneered by using layering or press-on techniques [7,8].

Veneering ceramic chipping may be related to factors such as the non-anatomic design of the zirconia framework, inadequate bond strength, and increased veneering ceramic thickness [9], and it has been a commonly encountered complication [10,11,12]. Another factor that may contribute to chipping is the difference in thermal expansion coefficient between the framework material and the veneering ceramic [13] as residual tensile stresses are generated during cooling [6]. Fast cooling processes, which increases tensile stresses, have been widely preferred in dental laboratories while fabricating veneered zirconia prostheses [14]; thus, lower cooling rates and slow cooling processes have been recommended to reduce the residual stresses [6,8,15]. The heating rate was also associated with the internal stresses generated during the firing process [16]. However, the furnace is not the only component of the firing process as auxiliary components such as firing trays and firing substrates are also a part of this process. These auxiliary components may be particularly critical while fabricating esthetic restorations such as laminate veneers, which can still be fabricated with stacking, even though digital technologies have expanded the possible fabrication methods [17,18].

A recent study has reported the significant effect of different firing tray systems on the residual stresses of a millable ceramic [19]. However, knowledge on how mechanical properties such as density, hardness, and biaxial flexural strength are affected when these ceramics are fired over different firing trays and firing substrates is limited. Veneered zirconia prostheses are still preferred in the daily clinical routines of both clinicians and dental technicians, and a study based on the mechanical properties of zirconia veneering ceramics when fired by using different firing trays and firing substrates may introduce different auxiliary components that could potentially reduce mechanical complications and increase the fit of esthetic restorations. Therefore, the present study aimed to evaluate the effect of different firing trays and firing substrates on the density, shrinkage, biaxial flexural strength, Martens’ hardness, and elastic indentation modulus of two veneering ceramics with different fusing temperatures. The null hypotheses were that ceramic type, firing tray, and firing substrate would not affect the (i) density, (ii) shrinkage, (iii) biaxial flexural strength, (iv) Martens’ hardness, and (v) elastic indentation modulus of zirconia veneering ceramics.

## 2. Materials and Methods

### 2.1. Specimen Preparation

The particle size distribution of the tested veneering ceramics was evaluated before the study by using a laser particle sizer (Laser Particle Sizer ANALYSETTE 22 MicroTec Plus; Fritsch GmbH, Idar-Oberstein, Germany) in wet dispersion with integrated green (523 nm) and infrared (940 nm) lasers. A total of 240 disk-shaped specimens (∅ 15 mm × 1.8 mm) were fabricated by using either a high-fusing (zirkon HFZ [HFZ]; estetic ceram ag, Triesen, Liechtenstein) or low-fusing (structure [STR]; estetic ceram ag, Triesen, Liechtenstein) zirconia veneering ceramic (Table 1) (N = 120).

**Figure 1:**
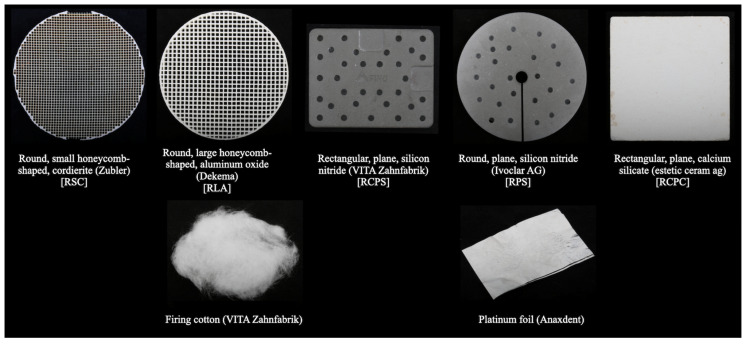
Firing trays and firing substrates tested in this study.

**Figure 2:**
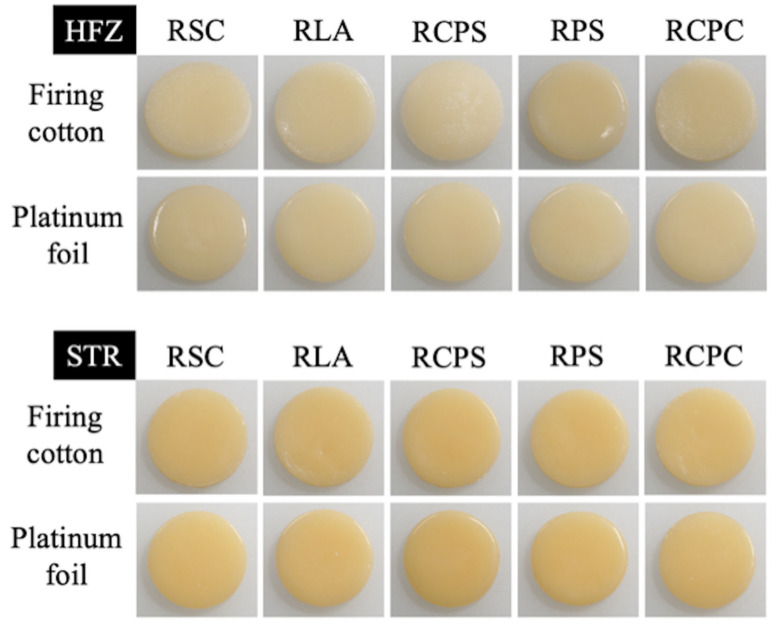
Representative images of one specimen from each firing tray–firing substrate pair within each zirconia veneering ceramic. RCPC, Rectangular, plane, calcium silicate; RCPS, Rectangular, plane, silicon nitride; RLA, Round, large honeycomb-shaped, aluminum oxide; RPS, Round, plane, silicon nitride; RSC, Round, small honeycomb-shaped, cordierite.

A stamp mold was used to fabricate the specimens, and the mold was isolated with an insulating pen (VITA Modisol; VITA Zahnfabrik, Bad Säckingen, Germany) to ensure a defect-free release. The ceramic powder (0.75 g) was mixed with a modeling liquid (0.25 g, estetic ceram ag) and applied into the mold with a brush (Genius; Renfert, Hilzingen, Germany). The ceramic was compacted by tapping the mold and soaking up the excessing liquid with paper tissues. The specimens were then removed from the mold and divided into 10 subgroups (n = 12) according to the firing tray and firing substrate pair they were fired on (Figure 1). All specimens (Figure 2) were fired in the same ceramic furnace (Austromat 654; Dekema, Freilassing, Germany) according to the manufacturer’s recommended firing parameters (Table 2). No pins were used during the firing process to avoid the distortion of the disk-shaped specimens, and the specimens were positioned circularly around the edge of the firing tray to ensure a comparable distance to the furnace’s heating coil.

### 2.2. Density and Shrinkage

After firing, the density of each specimen was determined in deionized water according to Archimedes’ principle (NewClassic MS; Mettler Toledo, Columbus, OH, USA) by using an analytical scale. To evaluate the shrinkage, each specimen was scanned with a laboratory scanner (Ceramill Map 400; Amann Girrbach, Koblach, Austria) that has 6 µm of accuracy and its corresponding software program (Ceramill Mind v2.4; Amann Girrbach, Koblach, Austria) to generate their standard tessellation language (STL) files. An anti-reflective scan powder (Arti-Spray; Dr. Jean Bausch, Cologne, Germany) was used before the scans to avoid light scattering from the specimens’ surfaces. These STLs were imported into a customized software program (QualityCheck; r2 dei ex machina GmbH, Remchingen, Germany), and the shrinkage of each specimen was automatically calculated by the software program after green state dimensions were specified (n = 12).

Each specimen was ground to a final thickness of 1.2 ± 0.05 mm and a final diameter of 12 mm with a polishing machine (Abramin; Struers, Ballerup, Denmark) and diamond grinding pads (40 µm and 20 µm, MD Rondo; Struers, Ballerup, Denmark) under constant water irrigation. Each specimen was then polished to high-gloss with polishing pads (MD Largo; Struers, Ballerup, Denmark) and diamond suspensions (DiaPro Largo 3 µm and 9 µm; Struers, Ballerup, Denmark). Finally, all specimens were ultrasonically cleaned (DT 31 H; Bandelin, Berlin, Germany) in 96% ethanol (Otto Fischer) for 3 min. The final thickness of each specimen was controlled with an electronic micrometer (Wabeco, Remscheid, Germany).

### 2.3. Flexural Strength

The biaxial flexural strength was measured by using a piston-on-3-ball setup (n = 12). The balls (∅3.2 mm, hardened steel) were arranged on a metallic platform in an equilateral triangle. The specimens were placed on the balls (support circle ∅10.2 mm) concentrically under the piston (∅1.6 mm, hardened steel). The load was applied at 1 mm/min crosshead speed until fracture by using a universal testing machine (Zwick 1445İ Zwick-Roell, Ulm, Germany). The flexural strength test was performed according to the International Organization for Standardization (ISO) standard 6872, and the flexural strength was calculated with recorded fracture load by using the following formula [20]:

*σ_f_* = −0.2387 *P* (X − Y)/*d*^2^

X = (1 + *v*) *In*(*r*_2_/*r*_3_)^2^ + [(1 − *v*)/2] (*r*_2_/*r*_3_)^2^

Y = (1 + *v*) [1 + *In*(*r*_1_/*r*_3_)^2^] + (1 − *v*) (*r*_1_/*r*_3_)^2^

where *σ*: flexural strength (MPa); *P*: fracture load (N); *d*: thickness (mm); *v*: Poisson’s ratio for veneering ceramics (0.19) [21]; *r*_1_: radius of the support circle (mm); *r*_2_: radius of the piston (mm); and *r*_3_: radius of the specimen (mm).

### 2.4. Martens’ Hardness and Indentation Modulus

Martens’ hardness (*HM*) and the indentation modulus (*E_IT_*) were determined by using a universal hardness testing machine (ZHU 0.2; Zwick-Roell, Ulm, Germany) (n = 12). The specimens were loaded with 9.81 N for 10 s with a Vickers diamond indenter (α = 136°). Within the testing software program (testX-pertV12.3 Master; Zwick-Roell, Ulm, Germany), *HM* and *E_IT_* were calculated using the following formulas [22]:

$$HM=\frac{F}{A_{s}(h)}$$

where *HM* = Martens’ hardness (N/mm^2^); *F* = applied load (N); and *A_s_*(*h*) = penetrated area of indenter at distance h from tip to specimen surface (mm^2^).

$$E_{IT}=(1-\upsilon_{S}^{2}){\left(\left(2\sqrt{\frac{A_{P}\left(h_{C}\right)}{\sqrt{\pi }S}}\right)-\left(\frac{1-\upsilon_{i}^{2}}{E_{i}}\right)\right)}^{-1}$$

where *E_IT_* = elastic indentation modulus (N/mm^2^); *A_p_*(*h_c_*) = projected contact area at loading (mm^2^); *v* = Poisson’s ratio of specimen and indenter with *v_S_* = 0.19 [21] and *v_i_* = 0.3; and *S* = contact stiffness derived from force removal curve.

### 2.5. Statistical Analysis

The Shapiro–Wilk test was used to evaluate the data distribution within each parameter. Given that data were distributed normally within each parameter, a generalized linear model, which included ceramic type, firing tray, and firing substrate as main factors, and every possible interaction, was used to further evaluate the data. A statistical analysis software program (SPSS v23; IBM Corp, Seattle, WA, USA) was used to evaluate the data with a significance level set at α = 0.05.

## 3. Results

**Figure 3:**
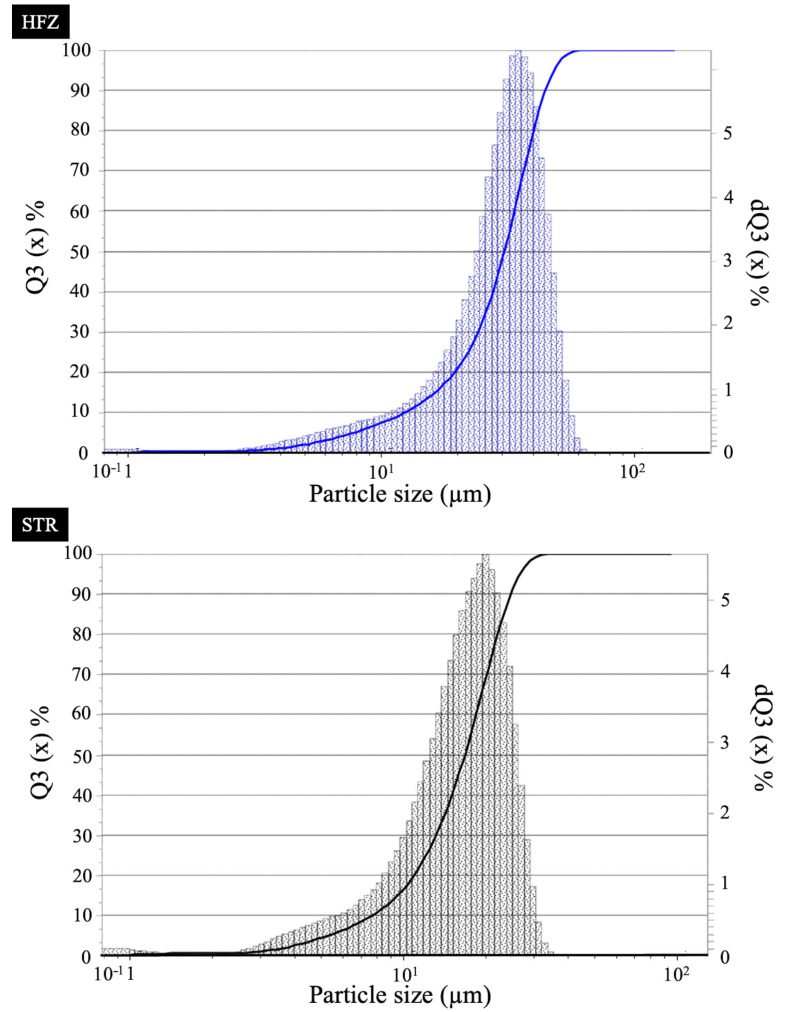
Particle size distribution for HFZ and STR. HFZ, zirkon HFZ; STR, structure.

The powder particle size distribution was calculated as D10 = 6.3 µm, D50 = 29.6 µm, and D90 = 56.2 µm µm for HFZ and as D10 = 3.9 µm, D50 = 16.7 µm, and D90 = 34.6 µm for STR (Figure 3).

Ceramic type, firing substrate, and the interaction between the ceramic type and the firing tray affected the density of the specimens (*p* ≤ 0.007) (Table 3). Regardless of the firing tray, HFZ (2.512 ± 0.010) had higher density than STR (2.440 ± 0.017) (*p* < 0.001), and platinum foil (2.478 ± 0.037) resulted in higher density than firing cotton (2.474 ± 0.040) (*p* = 0.007). HFZ-RSC and HFZ-RLA had higher density than HFZ-RCPS (*p* ≤ 0.028). STR-RSC and STR-RLA had lower density than the remaining STR pairs (*p* ≤ 0.046). In addition, STR-RCPS had higher density than STR-RCPC (*p* = 0.048) (Table 4).

The main factors and every interaction other than those between the ceramic type and the firing substrate (*p* = 0.115), and the firing tray and the firing substrate (*p* = 0.112), affected the shrinkage of tested specimens (*p* ≤ 0.012) (Table 3). Regardless of the firing substrate–firing tray pair, STR had higher shrinkage (*p* < 0.001). RSC led to lower shrinkage than RLA within HFZ–firing cotton, to the lowest shrinkage within HFZ–platinum foil, and to lower shrinkage than RCPC within STR–firing cotton (*p* ≤ 0.045). Platinum foil led to lower shrinkage within HFZ-RSC and HFZ-RCPC (*p* ≤ 0.004) (Table 5).

The generalized linear model analysis revealed that the ceramic type, firing substrate, and the interaction among all main factors affected the biaxial flexural strength values (*p* ≤ 0.045) (Table 3). STR had higher flexural strength within the firing cotton–RSC pair (*p* = 0.002). Within HFZ–firing cotton, RCPS led to higher flexural strength than RCPC (*p* = 0.034), and within HFZ–platinum foil, RCPC led to lower flexural strength than RSC, RCPC, and RLA (*p* ≤ 0.045). RSC led to higher flexural strength within STR–firing cotton than RCPS and RCPC (*p* ≤ 0.017). Platinum foil led to higher flexural strength within HFZ-RSC and HFZ-RCPC (*p* ≤ 0.018) (Table 6).

The interaction between the firing tray and the firing substrate, and the interaction among all main factors affected the Martens’ hardness values (*p* ≤ 0.019) (Table 3). STR had higher Martens’ hardness within firing cotton–RLA and firing cotton–RPS (*p* ≤ 0.034), and HFZ had higher Martens’ hardness within firing cotton–RCPC (*p* = 0.003). Within HFZ–firing cotton, RCPC led to the highest and RPS led to the lowest Martens’ hardness (*p* ≤ 0.031). Within STR–firing cotton, RLA led to the highest Martens’ hardness (*p* ≤ 0.026). Firing cotton led to higher Martens’ hardness within HFZ-RCPC and STR-RLA, and platinum foil led to higher Martens’ hardness within HFZ-RPS (*p* ≤ 0.011) (Table 7).

The indentation modulus of the specimens was affected by the ceramic type, the interaction between the firing tray and firing substrate, and the interaction among all main factors (*p* ≤ 0.005) (Table 3). HFZ had a higher indentation modulus within firing cotton–RCPC (*p* = 0.007) and STR had a higher indentation modulus within firing cotton–RLA, firing cotton–RPS, and platinum foil–RSC (*p* ≤ 0.012). Within HFZ–firing cotton, RPS led to the lowest indentation modulus (*p* ≤ 0.002), and RCPC led to a higher indentation modulus than RSC and RLA (*p* ≤ 0.016). Within HFZ–platinum foil, RPS led to a higher indentation modulus than RSC (*p* = 0.030). Within STR–firing cotton, RLA led to the highest indentation modulus (*p* ≤ 0.043). Firing cotton led to a higher indentation modulus within HFZ-RCPS, HFZ-RCPC, and STR-RLA (*p* ≤ 0.044) (Table 8).

## 4. Discussion

The present study aimed to evaluate how ceramic type, firing tray, and firing substrate affected the density, shrinkage, biaxial flexural strength, Martens’ hardness, and elastic indentation modulus of zirconia veneering ceramics. The tested outcomes were affected by the interaction between or among independent variables. Therefore, all null hypotheses were rejected.

HFZ had higher density than STR, regardless of the firing tray and firing substrate, with a maximum mean difference of 0.080 g/cm^3^ (RLA). When the firing trays were compared, RSC and RLA led to higher density than RCPS within HFZ and led to the lowest density within STR. Therefore, it can be hypothesized that RSC and RLA may be more suitable while firing HFZ rather than STR. Nevertheless, the maximum mean difference among tested trays within each material was even smaller: 0.008 g/cm^3^ for HFZ and 0.015 g/cm^3^ for STR. These statistically significant differences may not have clinically perceptible outcomes, which should be substantiated with in vivo studies. The higher volumetric shrinkage of STR than that of HFZ may be related to the difference in the particle size distribution of the tested ceramics, and dental technicians may need to veneer a greater amount of STR to the occlusal or interproximal surfaces of prostheses than that of HFZ. Firing trays affected the volumetric shrinkage of HFZ regardless of the firing substrate, and that of STR when fired over firing cotton. However, the maximum meaningful differences within ceramics fired over firing cotton was 1.9% for HFZ and 2.3% for STR, and firing the tested ceramics over firing cotton may not have a clinically significant effect. Nevertheless, veneering is an operator-related process and the volumetric shrinkage of a prosthesis may differ from that of a disk-shaped specimen. For the HFZ–platinum foil pair, RSC led to the lowest volumetric shrinkage, and this firing tray may lead to less material usage. A similar interpretation can also be made for platinum foil while firing HFZ over RSC and RCPC trays when compared with firing cotton. The lesser shrinkage of HFZ over these firing trays and platinum foil may improve the internal and marginal fit of laminate veneers fabricated on platinum foil-covered refractory dies.

STR had higher biaxial flexural strength than HFZ only when the specimens were fired over RSC–firing cotton. When firing trays were considered, RCPS within HFZ and RSC within STR led to biaxial flexural strength values that were either similar to or higher than the other trays when firing cotton was used. However, when platinum foil was used, RCPS led to biaxial flexural strength values that were either similar to or lower than the other trays. When firing substrates were considered, platinum foil led to higher flexural strength values within HFZ when RSC and RCPC were used. Nevertheless, all specimens had biaxial flexural strength values higher than 50 MPa, which was indicated as the threshold value for a ceramic to be used for veneering crown frameworks or for the fabrication of an adhesively cemented crown, laminate veneer, inlay, or onlay in the anterior region by ISO 6872:2019 [20]. However, the present study investigated the initial biaxial flexural strength of the tested specimens, and future studies that involve the thermomechanical aging of the tested veneering ceramics are needed to elaborate the effect of ceramic type, firing tray, and firing substrate on the biaxial flexural strength and possible complications that might be encountered in the long term.

The impact of ceramic type on Martens’ parameters of tested zirconia veneering ceramics, when evaluated with specific firing trays and substrates, was relatively minor. These ceramics could potentially exhibit similar behavior in response to external stresses that are influenced by Martens’ parameters. In addition, considering that the Martens’ parameters of tested veneering ceramics were mostly similar, the frequency of possible esthetic and mechanical complications related to wear and force application could also be similar. Firing HFZ over RCPC and STR over RLA while using firing cotton may reduce the risks of complications as these combinations led mostly to higher Martens’ parameters. However, while using platinum foil, the effect of firing trays was minimized within the tested ceramics as only RPS led to a higher indentation modulus than RSC within HFZ. Using firing cotton while firing HFZ over RCPC and STR over RLA, and using platinum foil while firing HFZ over RPS may be beneficial to reduce the potential complications over time.

The authors are aware of only one study that evaluated how different firing trays affected the properties of ceramics [19]. Wendler et al. [19] investigated the effect of firing trays on the residual stresses of a millable zirconia-reinforced lithium silicate glass ceramic. Even though the outcomes of the present and Wendler et al.’s [19] studies were different, parallel findings were reported as that study showed that placing the prosthesis over a fibrous pad or firing paste significantly reduced the residual stresses when compared with placing it over a platinum, cordierite, or silicon nitride pin. In addition, the authors [19] emphasized the importance of extending the cooling protocol, which results in homogenous temperature distribution that prevents the development of residual stresses and premature failures.

A limitation of the present study was that an a priori power analysis could not be performed, given that the present study was the first to focus on the density, shrinkage, biaxial flexural strength, Martens’ hardness, and elastic indentation modulus of zirconia veneering ceramics with different final firing temperatures when fired by using different firing trays and firing substrates. Nevertheless, significant differences were observed within each parameter investigated; thus, the authors think that the number of specimens in each group was sufficient. Only two veneering ceramics from a single manufacturer were tested, which was another limitation of this study. In addition, the present study focused on the inherent properties of the tested veneering ceramics; thus, no aging was performed and how the tested ceramics behave in the long term could not be simulated. The absence of a zirconia framework is another limitation of this study; however, this approach was deliberately chosen to evaluate the inherent properties of the tested veneering ceramics. Disk-shaped specimens were tested in the present study to adhere to the tests of the evaluated properties. However, the mechanical and optical behavior of disk-shaped specimens and actual prostheses may differ. The present study used one ceramic furnace to fabricate the specimens by using standardized firing parameters recommended by the manufacturer, and no repetitive firing regimens were performed. Therefore, the findings of the present study should be corroborated by future studies that investigate the tested parameters when the specimens are fired by using different parameters or subjected to repetitive firing, and also investigate other clinically relevant properties such as bond strength and long-term resistance to physical stresses when the tested ceramics are veneered over a zirconia framework.

## 5. Conclusions

Firing trays and firing substrates affected the mechanical properties of the tested high- and low-fusing zirconia veneering ceramics. High-fusing veneering ceramics mostly had higher mechanical properties than the low-fusing veneering ceramics. Firing the tested veneering ceramics over a round and small honeycomb-shaped cordierite firing tray and firing cotton mostly led to higher mechanical properties. Nevertheless, all specimens had flexural strength higher than 50 MPa, which is the ISO-specified value for veneering ceramics of crown frameworks or adhesively cemented monolithic single-unit restorations in the anterior region.

## Figures and Tables

**Table 1:** Zirconia veneering ceramics tested in this study.

Veneering Ceramic	Chemical Composition	Manufacturer	LOT Number
Zirkon HFZ (HFZ)	Silicon dioxide, aluminum oxide, potassium oxide, sodium oxide, calcium oxide, boron trioxide	estetic ceram ag, Triesen, Liechtenstein	260819
Structure (STR)	Silicon dioxide, aluminum oxide, potassium oxide, sodium oxide, lithium oxide, strontium oxide, boron trioxide, cerium(IV) oxide, zinc oxide		270620

**Table 2:** Firing parameters of tested zirconia veneering ceramics.

HFZ (zirkon HFZ)	450°C→45°C/min960°C1min Vacuum level of 100% at 960°C, 1 min
STR (structure)	450°C→45°C/min800°C1min Vacuum level of 100% at 800°C, 1 min

**Table 3:** Results of generalized linear model analysis within each parameter.

	Density	Shrinkage	Biaxial Flexural Strength	Martens’ Hardness	Indentation Modulus
Ceramic type	<0.001	<0.001	0.025	0.284	0.002
Firing tray	0.391	<0.001	0.127	0.197	0.195
Firing substrate	0.007	<0.001	0.016	0.212	0.157
Ceramic type × Firing tray	<0.001	0.012	0.227	0.060	0.060
Ceramic type × Firing substrate	0.054	0.115	0.073	0.896	0.362
Firing tray × Firing substrate	0.521	0.112	0.313	0.001	<0.001
Ceramic type × Firing tray × Firing substrate	0.064	0.004	0.045	0.019	0.005

**Table 4:** Mean ± standard deviation density (g/cm^3^) values of each material–firing tray pair.

Firing Tray	HFZ (Zirkon HFZ)	STR (Structure)
RSC	2.514 ± 0.009 ^Bb^	2.435 ± 0.013 ^Aa^
RLA	2.514 ± 0.010 ^Bb^	2.434 ± 0.017 ^Aa^
RCPS	2.506 ± 0.010 ^Ab^	2.449 ± 0.023 ^Ca^
RPS	2.512 ± 0.010 ^ABb^	2.444 ± 0.010 ^BCa^
RCPC	2.512 ± 0.005 ^ABb^	2.442 ± 0.014 ^Ba^

Different superscript uppercase letters indicate significant differences in columns and different superscript lowercase letters indicate significant differences in rows (*p* < 0.05).

**Table 5:** Mean ± standard deviation volumetric shrinkage (%) values of each firing substrate–firing tray pair within tested materials.

Material	Firing Tray	Firing Cotton	Platinum Foil
Z (zirkon HFZ)	RSC	31.75 ± 2.70 ^Ab#^	26.65 ± 2.71 ^Aa#^
	RLA	33.65 ± 5.45 ^Ba#^	32.43 ± 4.19 ^Ba#^
	RCPS	32.21 ± 2.26 ^ABa#^	31.82 ± 1.17 ^Ba#^
	RPS	32.61 ± 3.60 ^ABa#^	32.84 ± 2.39 ^Ba#^
	RCPC	33.56 ± 2.47 ^ABb#^	30.81 ± 1.23 ^Ba#^
S (structure)	RSC	36.98 ± 1.85 ^Aa^*	37.17 ± 1.35 ^Aa^*
	RLA	38.03 ± 1.24 ^ABa^*	37.39 ± 1.73 ^Aa^*
	RCPS	38.75 ± 1.20 ^ABa^*	37.83 ± 1.36 ^Aa^*
	RPS	38.79 ± 1.64 ^ABa^*	37.46 ± 1.47 ^Aa^*
	RCPC	39.30 ± 1.93 ^Ba^*	37.51 ± 1.15 ^Aa^*

Different symbols indicate significant differences among materials within each firing substrate–firing tray pair. Different superscript uppercase letters indicate significant differences in columns within each ceramic–firing substrate pair and different superscript lowercase letters indicate significant differences in rows within each ceramic–firing tray pair (*p* < 0.05).

**Table 6:** Mean ± standard deviation biaxial flexural strength (MPa) values of each firing substrate–firing tray pair within tested materials.

Material	Firing Tray	Firing Cotton	Platinum Foil
Z (zirkon HFZ)	RSC	72.02 ± 5.84 ^ABa#^	90.25 ± 11.29 ^Bb^*
	RLA	85.67 ± 9.29 ^ABa^*	93.57 ± 16.89 ^Ba^*
	RCPS	90.43 ± 5.36 ^Ba^*	79.87 ± 20.08 ^Aa^*
	RPS	78.94 ± 12.85 ^ABa^*	84.64 ± 10.55 ^ABa^*
	RCPC	67.57 ± 12.45 ^Aa^*	90.30 ± 11.57 ^Bb^*
S (structure)	RSC	95.40 ± 13.80 ^Ba^*	91.66 ± 13.19 ^Aa^*
	RLA	90.41 ± 14.64 ^ABa^*	90.06 ± 12.45 ^Aa^*
	RCPS	81.01 ± 5.79 ^Aa^*	86.95 ± 13.03 ^Aa^*
	RPS	88.74 ± 16.71 ^ABa^*	90.04 ± 7.22 ^Aa^*
	RCPC	81.31 ± 8.72 ^Aa^*	84.58 ± 8.54 ^Aa^*

Different symbols indicate significant differences among materials within each firing substrate–firing tray pair. Different superscript uppercase letters indicate significant differences in columns within each ceramic–firing substrate pair and different superscript lowercase letters indicate significant differences in rows within each ceramic–firing tray pair (*p* < 0.05).

**Table 7:** Mean ± standard deviation Martens’ hardness (N/mm^2^) values of each firing substrate–firing tray pair within tested materials.

Material	Firing Tray	Firing Cotton	Platinum Foil
Z (zirkon HFZ)	RSC	2259 ± 481 ^Ba^*	2228 ± 379 ^Aa^*
	RLA	2373 ± 326 ^Ba#^	2133 ± 342 ^Aa^*
	RCPS	2384 ± 415 ^Ba^*	2197 ± 367 ^Aa^*
	RPS	1901 ± 486 ^Aa#^	2418 ± 328 ^Ab^*
	RCPC	2724 ± 272 ^Cb^*	2322 ± 462 ^Aa^*
S (structure)	RSC	2249 ± 496 ^Aa^*	2328 ± 442 ^Aa^*
	RLA	2707 ± 274 ^Bb^*	2225 ± 406 ^Aa^*
	RCPS	2356 ± 415 ^Aa^*	2304 ± 466 ^Aa^*
	RPS	2317 ± 383 ^Aa^*	2366 ± 416 ^Aa^*
	RCPC	2247 ± 420 ^Aa#^	2374 ± 406 ^Aa^*

Different symbols indicate significant differences among materials within each firing substrate–firing tray pair. Different superscript uppercase letters indicate significant differences in columns within each ceramic–firing substrate pair and different superscript lowercase letters indicate significant differences in rows within each ceramic–firing tray pair (*p* < 0.05).

**Table 8:** Mean ± standard deviation indentation modulus (N/mm^2^) values of each firing substrate–firing tray pair within tested materials.

Material	Firing Tray	Firing Cotton	Platinum Foil
Z (zirkon HFZ)	RSC	47 ± 7.2 ^Ba^*	43 ± 5.6 ^Aa#^
	RLA	48 ± 6.4 ^Ba#^	44 ± 5.3 ^ABa^*
	RCPS	49 ± 5.6 ^BCb^*	44 ± 6.3 ^ABa^*
	RPS	39 ± 6.9 ^Aa#^	49 ± 7.0 ^Bb^*
	RCPC	53 ± 5.8 ^Cb^*	46 ± 6.9 ^ABa^*
S (structure)	RSC	47 ± 7.8 ^Aa^*	50 ± 8.0 ^Aa^*
	RLA	55 ± 4.6 ^Bb^*	47 ± 6.8 ^Aa^*
	RCPS	50 ± 8.2 ^Aa^*	47 ± 6.7 ^Aa^*
	RPS	47 ± 5.2 ^Aa^*	50 ± 6.2 ^Aa^*
	RCPC	46 ± 7.0 ^Aa#^	49 ± 6.9 ^Aa^*

Different symbols indicate significant differences among materials within each firing substrate–firing tray pair. Different superscript uppercase letters indicate significant differences in columns within each ceramic–firing substrate pair and different superscript lowercase letters indicate significant differences in rows within each ceramic–firing tray pair (*p* < 0.05).

## Data Availability

Data are contained within the article.
